# Voluntary stopping of eating and drinking (VSED) as an unknown challenge in a long-term care institution: an embedded single case study

**DOI:** 10.1186/s12912-018-0309-8

**Published:** 2018-09-01

**Authors:** Nadine Saladin, Wilfried Schnepp, André Fringer

**Affiliations:** 1 Institute of Applied Nursing Science IPW-FHS, FHS St.Gallen, University of Applied Sciences, Rosenbergstrasse 59, 9001 St. Gallen, Switzerland; 2 Faculty of Health, Department of Nursing Science, Chair of Family Oriented and Community Based Care, Witten/Herdecke University, Stockumer Strasse 12, 58453 Witten, Germany; 3 Institute of Nursing, School of Health Professions, ZHAW Zurich University of Applied Sciences, Technikumstr. 81, CH-8400 Winterthur, Switzerland

**Keywords:** Embedded single case study, Voluntary stopping of eating and drinking (VSED), Hastened death, Unbearable suffering, Long-term care, Palliative care, Nurses, Relatives

## Abstract

**Background:**

Chronically ill persons experience conditions of life that can become unbearable, resulting in the wish to end their life prematurely. Relatives confronted with this wish experience ambivalence between loyalty to the person’s desire to die and the fear of losing this person. Caring for a person during the premature dying process can be morally challenging for nurses. One way to end one’s life prematurely is Voluntary Stopping of Eating and Drinking (VSED).

**Methods:**

This embedded single case study explored the experiences of registered nurses (embedded units of analysis: ward manager, nursing manager, nursing expert) and relatives who accompanied a 49-year-old woman suffering from multiple sclerosis during VSED in a Swiss long-term care institution (main unit of analysis). By means of a within-analysis, we performed an in-depth analysis of every embedded unit of analysis and elaborated a central phenomenon for each unit. Afterwards, we searched for common patterns in a cross-analysis of the embedded units of analysis in order to develop a central model.

**Results:**

The following central concept emerged from cross-analysis of the embedded units of analysis: As a way of ending one’s life prematurely, VSED represents an unfamiliar challenge to nurses and relatives in the field of tension between one’s personal attitude and the agents' concerns, fears and uncertainties. Particularly significant is the personal attitude, influenced on the one hand by oneˊs own experiences, prior knowledge, role and faith, on the other hand by the VSED-performing person's age, disease and deliberate communication of the decision. Depending on the intention of VSED as either suicide or natural dying, an accepting or dismissing attitude evolves on an institutional and personal level.

**Conclusions:**

To deal professionally with VSED in an institution, it is necessary to develop an attitude on the institutional and personal level. Educational measures and quality controls are required to ensure that VSED systematically becomes an option to hasten death. As VSED is a complex phenomenon, it is necessary to include palliative care in practice development early on and comprehensively. There is a high need of further research on this topic. Particularly, qualitative studies and hypothesis-testing approaches are required.

## Background

Persons suffering from chronical diseases, e.g. multiple sclerosis, experience conditions of life which can become unbearable [1]. This may result in the wish to end one’s life prematurely [2, 3]. There a several options. One of them is assisted suicide which is legal in Switzerland, in contrast to many other countries [4]. Switzerland can be called a "right-to-die society" [5]. In 2014, 742 persons in Switzerland died by assisted suicide [6], accounting for 1.2% of all deaths [6]. 94% of these persons were older than 55 years, most of them suffering from a chronic disease [6].

Another way of ending one’s life prematurely is Voluntary Stopping of Eating and Drinking (VSED) [7–10]. This concerns cognitively unimpaired persons deliberately renouncing food and fluid with the aim to hasten death [7–10]. The definition of VSED applies only to persons being physically capable of oral food/fluid intake and digestion [7]. Additionally, it is important to distinguish VSED from declining interest in food and fluid in persons at the end of life [10, 11]. VSED is characterized by the discipline and endurance of the performing person [12]. Therefore, it is an volitional act, extended over a long time and not a situational impulse [12]. If chronically ill persons decide to end their life prematurely, mental, social and spiritual factors are relevant [13]. Nurses having cared for persons during VSED described various motives for deciding in favor of VSED [10]. They mentioned that the affected persons were willing to die, considered it senseless to go on living, had a low quality of life and wished to control the circumstances of dying [10]. As reasons for wishing a hastened death, affected persons named a deterioration of health status and progression of the disease [11, 14–16]. Furthermore, the burdens of life prevailed over reasons for continuing to live [11, 14–16]. They mentioned to be "tired of life" or to have done everything they wanted [11, 14–16]. Reasons for choosing VSED instead of other methods were related to the possibility of controlling the circumstances of one’s death and to act in a self-determined way [11, 14–16]. In planning to fulfill the intention to hasten death, persons often involve their relatives [17]. Attending persons can take over the task of arranging the process of dying as comfortable as possible [18]. This comprises symptom management, taking care of "last things" and saying goodbye [18]. A challenging situations occurs if the person performing VSED suffers from a delirium and wishes to drink [9]. In this case, Quill and Byock (2000) recommend to fulfill this wish [9]. If this is a recurrent problem, VSED should be reconsidered [9]. Chabot (2011) proposes to discuss this scenario in advance with the affected person and to determine how to proceed in this case [18]. Moreover, sedation should be taken into account [18]. Presumably, the accompanying symptom of thirst refers rather to xerostomia than to the desire to drink [18, 19]. Xerostomia can be treated by means of oral care [18, 19]. Due to oral care, persons having ceased to drink take in about 50 ml of fluid per day [20]. It is possible that persons withdraw from the decision for VSED [10]. Reasons for resuming food intake can be various [10]. Ganzini et al. (2003) mention, for example, pressure on the part of relatives, encouragement to resume food-intake, discomfort and hunger, diminished depression or alleviation of concerns [10]. During VSED, tiredness occurs and in a later stage loss of consciousness caused by an increased blood urea level [20]. Continuing to take in small amounts of fluid stimulates urea elimination via the kidneys [20]. This results in a prolonged dying process, however, it also allows intermittent periods of clear consciousness until shortly before death [20]. According to Chabot (2011), VSED lasts seven to 15 days until death occurs if fluid and food are stopped simultaneously [20]. In persons stopping only eating and reducing fluid-intake during several days or weeks, death is to be expected after 16 to 30 days [20]. According to Chabot and Goedhart (2009), death during seven days after stopping drinking can be attributed to the underlying disease or to medication [17]. Usually, persons die from VSED in deep-sleep, mostly caused by circulatory arrest due to dehydration or complications like pneumonia [7, 17, 20]. Attending persons describe death by VSED as peaceful and soft, without suffering or pain and with a pleased expression before death [10, 16, 21]. Nurses having cared for persons requesting premature death reported fears of offending the law. Thus, it seems important to clarify the legal situation [22]. From a legal point of view, VSED is an act of self-killing, although it does not consist of an action but an omission [23]. VSED is positioned between the personal freedom of every human being to decide how and when to end life and the duty of the state and every person to protect another human beingˊs life [24]. In case of withholding life-sustaining measures in a person willing to die, the right to autonomy is rated higher than the duty to sustain life [24].

The decision to end one's life prematurely can release several emotions in relatives, e.g. rejection, futility, co-responsibility and excessive demand [25]. Furthermore, thinking of an agonizing death caused by thirst can provoke fears [26]. Chabot (2011) describes the ambivalence experienced by relatives [18]. On the one hand, they want to stay loyal towards the affected person. On the other hand, they defend themselves against the fear of separation [18]. Feelings of guilt and anger towards the person wanting to die can also arise [18]. Eating means participating in social life [19]. Thus, relatives can misunderstand VSED as a rejection directed against them personally and as a decision against social participation [19]. According to Walther (2011), relatives respond to a person’s decision to die by feeling accountable with regard to insufficient support on their part [25]. This can lead either to more intensive support from relatives or to relieving relatives from support if they are already overburdened [25]. In contrast to other ways of self-killing, VSED allows relatives to mentally prepare for the forthcoming dying process [11, 21]. This offers the chance to clarify relationships possibly impacted by misunderstandings, disputes or conflicts [21]. Relatives' attitudes towards VSED have rarely been a subject of research. According to Chabot und Goedhart (2009), most relatives experience a family member's death by VSED as dignified [17]. VSED has been the subject of publications for a long time. As the literature shows, health care professionals are challenged in dealing with VSED [22]. For nurses, caring for persons during VSED comprises palliative care, informing and counselling [9]. Accompanying persons who decided to hasten death can be morally irritating for health care professionals [22]. They find themselves in the field of tension between a person's right to choose a hastened death and social, moral as well as mental aspects of valuable life [22]. Reflecting the implications of VSED and their own role proves to be important for them [17, 27]. Furthermore, nurses should be able to demarcate VSED from assisted suicide [17, 27].

Although nurses have no legal responsibility [9], Harvath et al. (2006) indicate that some of them feel personally responsible for the affected persons and their relatives [22]. A nurse reported that she had the feeling of having failed if patients decided for assisted suicide, since this expressed that they did not feel comfortable [22]. So far, nurses' attitudes towards VSED have been rarely researched. Harvath et al. (2006) described that nurses´ experience of caring for persons with VSED is less challenging than in the case of assisted suicide [22]. Nurses perceive VSED as a natural process, causing less emotional burden for relatives [22]. They also describe VSED as "letting go of life" [22]. In contrast, assisted suicide is an active, temporally limited action from the nurses' point of view [22]. However, health care professionals also express fear of enlarging suffering by means of VSED and thereby causing additional burden [19].

In 2015, the following incident happened in a Swiss long-term care institution. A 49-year old resident suffering from multiple sclerosis decided to end her life by VSED due to progressing mobility impairments and dependency for bowel voiding after an incurable exacerbation. In view of lacking professional experience with this method, the institution contacted an expert (AF) for VSED. In agreement with the institution (general manager, responsible for institutional business as well as for the public representation of the long-term care institution and project commission and the nurse manager, responsible for the staffed nurses as well as for quality and safety of nursing care), the expert team provided external support for the VSED process (especially for the nursing expert, who is responsible for the scientific questions of nursing practice). Against this background, the necessity of an in-depth investigation of this case arose (including the ward nurse, who is responsible for management of the ward, as well as nurses involved in the care of the resident). So far, there are only several case reports concerning VSED and a few studies exploring nurses' attitudes towards VSED. However, up to now, there is no qualitative comprehensive *case study research*, investigating a case from multiple perspectives. Additionally, individual suffering of chronically ill persons in the context of VSED also has hardly been researched so far.

### Aim

This study intended to comprehensively investigate the complexity of the VSED phenomenon from different perspectives. This is possible by means of an embedded single case study allowing to investigate various processes, attitudes and approaches, found to be necessary in the current case to explore the experiences of the persons involved [28].

### Research questions

Against this background, we derived the following research questions: What are the experiences of registered nurses, nurse managers, nurse experts and relatives in caring for a resident suffering from multiple sclerosis – from the first intention to choose VSED until death? What is the common pattern underlying the different embedded units of analysis within the case in dealing with the situation?

## Methods

Since this study investigates subjective experiences, we chose a qualitative design allowingto answer the research questions in a circular way [29].

### Design

To explore how the persons involved experienced the given situation, an embedded single case study is best suited [28, 30]. The origin of qualitative case studies lies in anthropology and sociology [29]. Merriam (1991) and Yin (2003) define a case study as an in-depth empirical inquiry of a contemporary phenomenon within its real-life context [30, 31]. Based on the given situation, it is possible to investigate the complexity of the VSED phenomenon from different perspectives aiming at a comprehensive understanding [32]. Individuals, groups or social interactions are defined for instance as units of analysis [33]. In the current study, the long-term care institution in which the 49-year-old woman suffering from multiple sclerosis and performed VSED represents the main unit of analysis. To this end, we assigned the persons involved into four groups and explored their experiences. This resulted in an embedded single case study design allowing to compile several units of analysis into one case [29, 33]. A unit lesser than the main unit of analysis is defined as an “embedded unit of analysis” [33]. In the current study, we identified four embedded units of analysis, analysed each unit (within-analysis) and then compared all embedded units of analysis in a cross-analysis. The embedded units of analysis of this study consist of the attending registered nurses, the ward manager, the nursing manager and the nursing expert as well as the relatives of the patient. The *bounded system* defining a case can consist of temporal or spatial aspects [29]. On a temporal level, the case in the current study includes the time between the idea of performing VSED until death. The case is temporally limited by the woman's death one year ago. The spatial limitation of the case relates to the long-term care institution where the woman lived, and she performed VSED.

### Sample

This study is based on a convenience sample since the initiative came from the long-term care institution (general manager and nurse manager). In the context of the given situation, we identified four criteria-related embedded units of analysis. The first unit consisted of eight nurses, the second of the ward nurse, the third of the nursing manager and the nursing expert and the fourth of the woman’s husband and son. They were also included as a unit of analysis because they were strongly affected by the decision for VSED and influenced the personal attitude of the nurses. In the end, the relatives were even more vulnerable than the person concerned and therefore had a strong impact on the nurses’ experiences with the VSED situation. The nursing expert with the gatekeeping function informed the nurses, the ward manager and the nursing manager about the study. Prior to the interviews, all participants received oral information about the significance, the scope and the consequences of participating in this study. We received oral informed consent and recorded it digitally. The gatekeeper also requested the relatives' interest in participation. After a positive response, the author contacted them by phone and explained the significance of the study as well as the implications of participating. Afterwards they received written information and an informed-consent formula.

### Data collection

According to Yin (2014), interviews are the primary data source of case studies [33]. Due to the explorative character of the research question, the interviews should rather have the form of a guided conversation than of a structured interview [33]. For this reason, we chose a narrative-generating approach for all four units of analysis. The interviews took place between February 2016 and December 2016.

Data-collection with registered nurses occurred by means of focus group interviews, representing the most recent form of interviews for a moderate group range [34]. Focus group interviews are appropriate to explore group experiences (coping process, dealing with the team, attitude of the team towards the topic of discussion). They are of interest as a method if several persons share similar experiences [34].

We performed a single interview with the ward manager and a group interview with the nursing manager and the nursing expert. Group interviews are suitable for two to three persons [34]. Finally, we conducted single interviews by phone with the husband and the son. We digitally recorded all interviews and transcribed them verbatim from Swiss dialect into German standard language, using transcription principles according to Flick (2009) in an adapted way [35]. During the whole research process we took field notes and wrote memos concerning methodological, personal and case-related issues. They serve to fix spontaneous thoughts and allow to fill the codes with meaning. By means of memos it is possible to grade and weigh the results [36].

### Data analysis

Data analysis occurred in two stages. First, we comprehensively analyzed every embedded unit of analysis (within-analysis) and afterwards performed a comparative analysis (cross-analysis). For within-analysis, Baker (2011) recommends to interpret data in a grounded theory style [37], i.e. in three steps [36]. The first step consists of open coding to disaggregate the text analytically [36]. This means that the interviews were read line by line and with constant comparison. The emerging open codes and in-vivo-codes were then bundled and allocated into broader generic codings. Afterwards, axial coding serves to refine and differentiate open codes [36] that were grouped into inductively developed sub-categories. The emerging category is positioned in the center, surrounded by a network of connections which have to be elaborated [36]. As a supporting instrument for the stages of axial and selective coding, a coding paradigm has to be developed. For the within-analysis, we identified the following axial categories: nursing and physician support during VSED, impact of VSED on the family, dimensions of VSED, and contextual factors. In the final stage of selective coding, we elaborated the central phenomenon of each embedded unit of analysis [36]. To this end, we re-worked existing codes, categories, memos and field notes until the central phenomenon emerged [36, 38]. Parts of the within-analysis are to be found again in the case description. At the same time, within-analysis represents the point of departure for cross-analysis of the embedded units of analysis.

We synthesized the results of the embedded units of analysis in the cross-analysis into one result [28]. As a supporting instrument, we used the word-table recommended by Yin (2014) to present axial codes of within-analysis in a homogeneous structure [33]. Finally, we analyzed all tables, searched for patterns and differences and drew conclusions for the units of analysis [33]. By comparing axial codes of the unit of nurses, the ward nurse, the nurse expert and the nurse manager, it was possible to elaborate the central phenomena for the individual axes of the coding paradigm. At this stage, the relatives served to understand the situation, decision and attitudes but were not involved in the cross-analysis. Therefore, the central phenomenon represents the professional situation in the case. On this basis, we developed a central model covering the central phenomenon of the professional units of analysis. Conclusively, all participants validated the results of our study by member-check. For data transcription, analysis and organisation we used MAXQDA 12 [39]. The presentation of the results follows a proposition from Cresswell (2013) [29].

### Trustworthiness

To ensure the trustworthiness of this study, we observed the quality criteria of credibility, transferability, reliability, transparency and authenticity [40]. We fulfilled these criteria by means of discussions within the research group (credibility), thick descriptions of all embedded units of analysis (transferability), review of the study by the second and last author (reliability and transparency) and by the embedded-single-case-study design, allowing in-depth exploration of experiences in the real-life-context (authenticity). The relatives of the person who chose VSED were also involved in the study to ensure a “convincing account” as one aspect of trustworthiness [41]. This was carried out by interviewing the relatives during the study process. The relatives and all other units of analysis (encompassing involved nurses, the ward nurse, the nursing expert and the nurse manager) were informed about the results after analysis completion – in form of a “member check”.

### Ethical aspects

The ethics committee of the canton St. Gallen reviewed the harmlessness of the study (EKSG16/016). All participants gave their written informed consent. In addition, also the oral informed consent was recorded digitally prior to data collection. Due to the risk of vulnerable situations emerging during the interviews, we informed participants about the possibility to end or interrupt the interview at any given time.

We irreversibly pseudonymised names of persons and places preventing conclusions with regard to institutions or persons. All participants received information about the aims, the procedure and a possible publication of the study. Additionally, we informed them about the possibility of withdrawing from the study at any time without consequences. The last author preserves the digital recordings.

## Results

In this section, we present the results of the qualitative data analysis, beginning with a description of the given situation. Afterwards, we outline all four embedded units of analysis and finally present the results of the cross-analysis.

### Situation

The affected person fell ill with multiple sclerosis 30 years ago. She had been living in the long-term care institution for three years. After an incurable exacerbation, she suffered from progressing mobility impairments. Transfers were possible but only by means of a patient lifter. Additionally, she experienced loss of strength in her hands, leading to impairments concerning eating and leisure activities as well as progressing dependency concerning intimate care and bowel voiding. These restrictions prevented her from continuing her usual visits at home during the weekend. She suffered from pain all over her body. Thus, she was confronted with a crisis and reflected her situation. Her suffering reached a point where she took the possibility into account of dying prematurely.

*“I think, she was not tired of life but simply tired of suffering.”* (NS&AF15022016_2 Z46, nurses)

The woman deliberately and voluntarily decided to stop eating in order to die prematurely and informed her relatives. This wish was not unexpected for them because she had thought about this option previously. The family accepted her wish, hoping she would choose assisted suicide. However, experiencing a dying process as consciously as possible was important for her. Hence, the family finally supported her request for VSED. In the middle of July, the family informed the nursing staff about the wish for VSED. The woman slowly started to reduce food and fluid intake. The nurses informed their superiors (ward manager and nursing expert) about the request for VSED and received the permission to start. When the nursing manager learned about VSED, she informed the general manager. In a conversation, the general manager told the family that VSED is not allowed in the institution. However, they would offer the possibility to organize outpatient care for performing VSED at home. The family did not understand why VSED was suddenly not allowed after obtaining the approval from the nursing expert. The option of performing VSED at home was not realistic for the woman. She preferred to be cared for by the nurses in the center. From the family’s point of view, VSED was not an act of self-killing. Therefore, they could not understand the interdiction and the general manager’s argumentation. Also for the nurses, the interdiction was incomprehensible. They accepted the woman’s wish and were able to understand it due to her long history of suffering and the progression of her illness. The ward manager, the nursing manager, the nursing expert and the nurses continued to advocate for the woman and supported her wish for VSED. They took measures in order to act against the interdiction, e.g. by submitting an application to the ethics committee or by writing a living will. The general manager on her part initiated to investigate the legal situation with regard to VSED. Finally, the nursing manager received the permission after having diagnosed urinary tract infection potentially leading to urosepsis with a probably fatal outcome. In view of this further deterioration and a psychiatric report excluding a depression, the general manager permitted to perform VSED in the centre. The nursing expert elaborated a plan to reduce food and fluid intake. To maintain "normality", the woman regularly attended all meals in the dining room and asked the nurses to remove beverages and meals without commentary. Every day she went to the cafeteria to drink an espresso with her husband. She had informed only one resident about performing VSED. In the further course, the nurses told all other residents that the woman’s condition had deteriorated further. The nurses described that the woman had changed after having made the decision. Prior to the decision, she often had been dissatisfied, whereas now she appeared to be relaxed and happy.

*“I did not know what to expect. I entered the room and I can still see it before my eyes: she was so radiant, as if…Yes, I was slightly irritated to see her so relaxed, happy and satisfied ...”* (NS&AF15022016_2 Z158, nurses)

While performing VSED, the woman deliberately expressed what she wanted to eat. At the beginning, it was a berry or a plum. At the end, she particularly liked flavoured ice cubes. She accurately controlled fluid-intake. While regarding how her body changed, she seemed to hope that the dying process would proceed faster. According to the nurses and her husband, she was impatient and could hardly await her death. She used the time to say goodbye to persons who were important to her. She visited them for the last time without telling them about VSED. Even a reconciliation with her daughter was possible after a conflict. According to the nurses, the terminal stage began approximately two weeks before her death. She became bedridden and refused fluid except for flavoured ice cubes. Until about ten days before her death she could clearly communicate, afterwards she used facial and vocal expression. During VSED, she received analgesics against headache and antiemetics against nausea. In the terminal stage, she additionally received morphine and lorazepam due to unrest. The nurses reported that at the end they had the impression of an inner struggle as she was very restless, and the terminal stage took a long time. She died in the middle of September, eight weeks after reducing food.

### Case description

In the following sections, we describe the identified embedded four units of analysis of this case study.

### Nurses

The participants consisted of nurses in training and registered nurses aged between 35 and 61 years with three to 33 years of professional experience. They worked on a ward for younger persons in need of care and had already accompanied the affected woman for three years before her decision for VSED. The woman herself had informed them about her wish and they cared for her until she died.

The nurses felt obliged to fulfill the woman's request and advocated for her:

*“…It is her will. We know her. So far, we have always cared for her. It was clear for us that she is completely competent. For me it is not a matter of judging what I personally think about this. It is her will and it is my task to support her”* (NS&AF15022016_1 Z49, nurses)

The nurses could not understand the attitude of the general manager since she barely knew the woman. Therefore, they tried to take action against the interdiction. Caring for the affected person during VSED was coherent for the nurses. Providing palliative care for her was not different from caring for persons dying naturally. The nurses reported that it was easy for them to care for the woman since she seemed to be happy.

The central phenomenon of the within-analysis is: *Struggling for the affected person in opposition to the management: respecting her request to die and being obliged to her wish*. This unit of analysis is significant to answer the research question since the nurses cared for the affected person during VSED and supported her request for VSED.

### Ward manager

The ward manager was a 61-year-old registered nurse with 33 years of professional experience. She also had cared for the woman for three years before the decision. During the performance of VSED, the ward manager accompanied the woman and her family and was the main contact person. During the whole time, she advocated for the woman, her family and the nurses. She represented their interests towards the management. In her eyes, VSED was something normal and legal. She did not understand the interdiction, considered the approach of the management to be non-transparent and felt uncomfortable with this situation. Caring for the woman during VSED was also coherent for her and she described the woman's death as dignified. The central phenomenon of the within-analysis is: *Being "in-between": ambivalence between promise and duty.* The ward managerˊs experience is important to answer the research question because she represented the interests of the affected person, the family and the nurses toward the management and at the same time felt responsibility towards the institution.

### Nursing manager and nursing expert

The nursing manager, 48 years, had 25 years of professional experience. The nursing expert, 51 years, possessed 30 years professional experience. The nursing expert was the first person involved in the case. She assumed the responsibility for professionally supporting the nurses. The nursing manager directly communicated with the general manager and mediated between her and the nurses. The nursing manager and the nursing expert ensured that the woman could perform VSED in the institution. However, they could also understand the concern and fear of the general manager. They tried to find a way that was possible and acceptable for all. The central phenomenon of the within-analysis of this unit of analysis is: *Moderating the situation and weighing the interests of persons involved: supporting the nurses and the family members to reach the aim to allow VSED in the institution*. This unit of analysis is important to answer the research question since both persons represent connecting links towards the management and tried to advocate for the interests of the affected woman and her relatives.

### Relatives (husband and son)

The family consists of the husband (62 years), the son (30 years) and the daughter (28 years). They accepted the mother's request for VSED and supported her. Every day, the husband spent time with his wife. Accompanying her was coherent for him and he described her death as beautiful and dignified. Although he suffered from the loss of his wife, he could understand her wish. The last time spent together during VSED and the death of his wife welded the family together. Accordingly, his relationship with his children had become very close. The son also visited his mother regularly during VSED. From his point of view, death relieved her from suffering. However, he would have wished for another way for his mother. Watching how her body changed during VSED was hardly bearable for him:

*“This was the most terrible moment, seeing her lying in bed, very emaciated and nearly irresponsive. This was a very, very terrible moment”* (NS12122016 Z12, relatives)

In his eyes, VSED is one of the most challenging ways of hastening death. However, he mentioned that his mother was a very willful woman doing everything to reach her aim and to have her will. The central phenomenon of the within-analysis of this unit of analysis is: *Caring and understanding: Respecting the request in spite of suffering due to illness and the wish to die.* This unit is meaningful to answer the research question since the relatives' experiences broaden the scope of the professional context, thereby offering a more comprehensive picture.

### Cross-analysis of embedded units of analysis

**Fig. 1:**
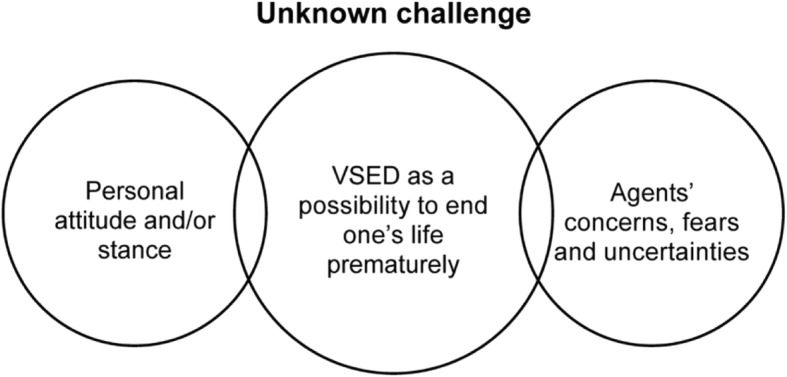
VSED as an unknown challenge in a long-term care institution

By means of a cross-analysis, it was possible to deduce a central model to answer the research question. The central concept summarizing the complexity of all units of analysis is presented in Fig. 1 and may be formulated this way: *VSED as an option of prematurely ending one's life represents an unknown challenge in the field of tension between one's personal attitude and the concerns, fears and uncertainties of the agents.*

**Fig. 2:**
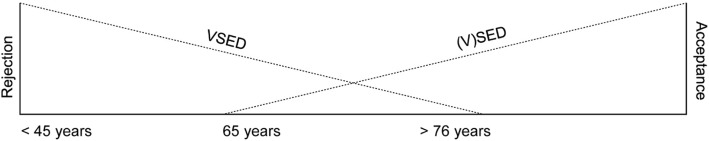
Development of an attitude towards VSED, depending on the age of the performing person

As the figure shows, VSED is at the centre as an option to end one's life. For all persons involved, VSED is unknown since there is rarely previous knowledge about this phenomenon and experience is lacking. Dealing with VSED is influenced by the personal attitude of every individual agent. Additionally, dealing with VSED as an unknown phenomenon evokes concerns, fears and uncertainties in all agents. The personal attitude towards VSED depends on the age of the performing person, as visible in Fig. 2.

This figure illustrates that the negative attitude towards VSED diminishes with increasing age of the performing person. With a certain age of the performing person, the acceptance rises. In younger persons, rejection of VSED seems to be highest. VSED is more accepted and perceived as a natural trajectory if the person is older.

*“In older persons you don’t want to think about the question if it is suicide or deliberate killing in this sense.”* (NS&AF15022016 Z92, nursing manager and nursing expert)

**Fig. 3:**
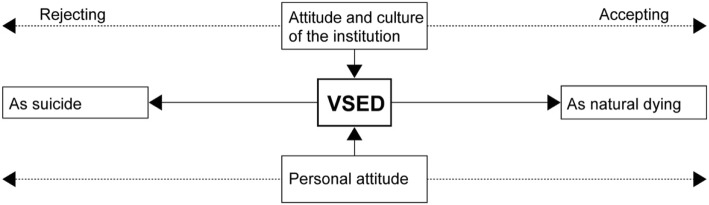
VSED in the field of tension between suicide and natural dying

As this figure shows, the attitude and culture of an institution is decisive for the way it deals with VSED. If VSED is interpreted as suicide, it is not allowed. Perceiving it as natural dying leads to acceptance and permission. This is also true on the personal level. A person interpreting VSED as suicide rejects it. Persons perceiving it as natural dying accept it. A combination of both models in Figs. 2 and 3 shows that implicit VSED in older persons is classified as natural dying and therefore accepted. In younger persons, however, VSED is interpreted as suicide resulting in rejection.

Aside of the age and the way of communicating VSED, other factors contribute to forming a personal attitude towards VSED: one's own experiences, prior knowledge, faith and role as well as the disease of the performing person. The attitude of the agents and the institution is particularly significant for dealing with VSED, as visible in Fig. 3.

**Fig. 4:**
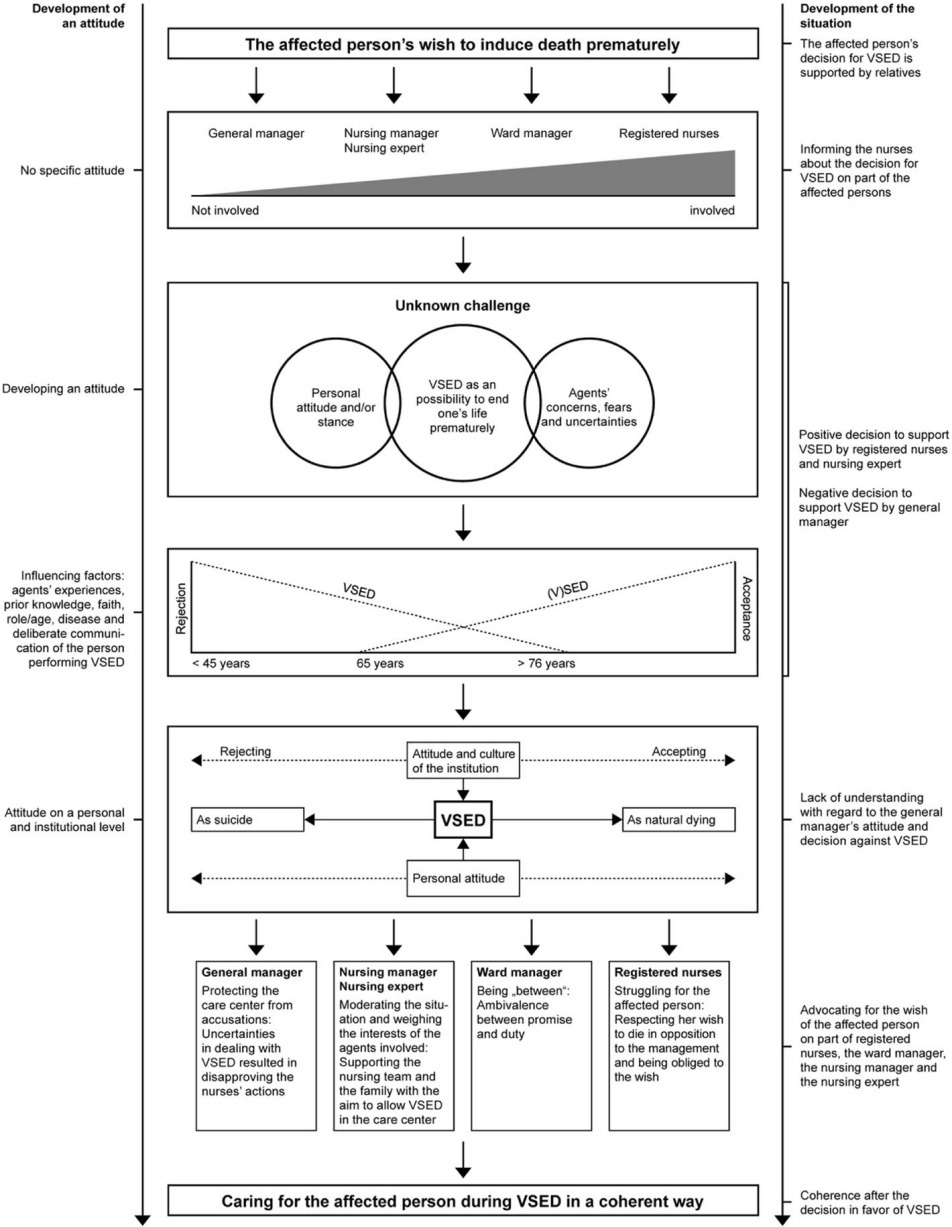
Central model: Dealing with VSED in long-term care institutions

The cross-analysis resulted in the model depicted in Fig. 4.

The decision of the woman and her relatives had an influence on all units of analysis and the general manager. In all agents, the process of developing an attitude began as described in Figs. 2 and 3. Due to different attitudes on the personal and institutional level, a conflict happened in the given situation. Against this background, there is a retroactive effect on the central phenomena of the units of analysis in the within-analysis. The result of this process was a form of caring for the affected woman that was perceived as coherent by all persons involved, allowing a dignified death, as expressed in the following quote:

*“Finally, a beautiful dying process. She found peace with her daughter and with herself and she was able to choose this process, to bring an end to this. And somehow was given a chance to leave this world as a human being. I’m still touched when I am thinking of her. Yes, I found it very beautiful that she chose this way and followed it so beautifully. So I personally think that it was a beautiful process.”* (NS&AF15022016_1 Z133, nurses)

## Discussion

For the first time, this embedded case study comprehensively investigated a situation of VSED from different perspectives of each of the group of caregivers and relatives. Based on the experiences of the participating persons, it was possible to elaborate a first model of dealing with VSED in a long-term care institution. The focus of this model reflects the major concept of this study: *VSED as an option of ending one’s life prematurely represents an unknown challenge in the field of tension between one's personal attitude and the concerns, fears and uncertainties of the agents.*

Furthermore, it became obvious that the age of the affected person directly influences the attitudes of the agents. Permission and performance of VSED in an institution significantly depend on the attitude towards VSED on the part of the individual agents and the institution. In the subsequent sections, we discuss the following central aspects: options of premature dying, challenges in caring for chronically ill persons, significance of personal attitude and coping with concerns and fears in a professional manner.

### Options of premature dying

Besides VSED, the literature describes three further methods of prematurely ending oneˊs life. The first is withholding life-sustaining interventions [21], the second death-accelerating analgesics and sedatives [20]. It is important to demarcate both methods from the killing on request since it is illegal in Switzerland [20]. Only nursing and palliative medical interventions leading to an accelerated dying process as a side effect are in accordance with the law [42, 43]. The third option consists of assisted suicide [20]. Persons who decided to end their life receive a lethal drug on medical prescription [20]. This method is not illegal in Switzerland and is offered by organisations like DIGNITAS or EXIT [20]. Although assisted suicide is legal, this was no option for the affected person as she wanted to ensure a natural dying process. Her son presumed that her faith might have played a role in this decision. In the woman's eyes, the drug prescribed for assisted suicide was poison. Poisoning herself was no option for her.

Classifying VSED proves to be difficult. Depending on the perspective, it can be regarded as withholding treatment, natural death or suicide [7]. Interpreted as an omission causing death, VSED can also be regarded as withholding treatment and therefore is a human right [7, 44]. Focusing on the deliberateness of the action, VSED can be classified rather as suicide [45, 46]. Wolfersdorf (1995) defines suicide as a self-induced action aiming to kill oneself [47]. This action is performed with the expectation and in the faith of achieving this aim by means of the chosen method [47].

However, VSED can be distinguished from suicide as the decision is reversible during the first days [16, 20]. To classify the wish to die, the person's current situation is significant [25, 48]. Is the person alive only by means of medical treatment, withholding treatment is not considered as suicide because it allows a natural death [25]. In this perspective, VSED can also be regarded as a form of withholding treatment [25]. In this context, Schwarz (2007) mentions that persons who are about to die from their disease, do not have the option to decide for life. As a consequence, VSED cannot be regarded as a decision against life [7].

The literature (clinical, philosophical, ethical and discipline-specific) offers heterogeneous answers to the question if VSED should be regarded as natural dying or suicide [49]. Concerning the woman's argument of not wanting to poison or kill herself, it can be assumed that from her point of view, VSED is not an act of self-killing.

The dying process in VSED corresponds to a natural dying process [20]. This distinguishes VSED from other forms of suicide [20]. The participating nurses confirm this view by comparing the dying process in VSED with the natural dying process.

For relatives, the difference between characterizing VSED as withholding treatment, natural death or suicide seems to be relevant on an emotional level [7]. Interpreting VSED as suicide can evoke pain, grief or anger and may negatively affect the bereavement process [7].

The elaborated model shows that classifying VSED as suicide or natural dying is important with regard to the way an institution deals with VSED. To ensure a professional way of dealing with VSED, the aim could consist in positioning VSED in the middle of a continuum ranging from suicide to natural dying. This may allow a reflected handling of VSED for all agents. Regarding VSED as suicide on a personal and institutional level leads to an interdiction of VSED without reflection. On the contrary, interpreting VSED as natural dying on the personal and institutional level, entails the danger of allowing VSED without reflection. This probably results in trivializing it since critical voices are absent. The results show that VSED in young persons is classified rather as a form of suicide, in contrast to implicitly renouncing eating and drinking in older persons. This indicates that institutions tend to reject VSED in younger persons, while implicit renouncement of eating and drinking in older persons is accepted and admitted without reflection. With regard to age-associated changes of food-intake, e.g. reduced appetite and feeling of thirst, swallowing problems, delirium or manual impairments [50], a non-reflected accepting attitude towards VSED can have potentially serious consequences, since the distinction between age-associated changes of food-intake and implicit renouncement of eating and drinking are not always clearly perceptible.

### Challenges in caring for chronically ill persons

Caring for chronically ill persons is marked by challenges [51]. It is not comparable with caring for acutely ill persons due to specific features of the nurse-patient-interaction. The aim does not consist in healing but in enabling persons to live with their illness and to preserve their quality of life [52]. The nursing role is extended by supporting, counselling and developing tasks [52]. The long-term patient-nurse-relationship leads to proximity [51]. This entails the danger of intermingling the professional and the everyday view [51]. In the situation described in this study it cannot be dismissed that the nurses' proximity to the affected woman had an influence on their personal view concerning VSED. The closer agents were to the affected woman, the greater was their endeavour to fulfil the woman's request. The nurses argued that they already had known the affected person for a long time and so they could comprehend her wish. The general manager was accused of deciding without knowing the woman and her situation.

Achieving a professional balance between proximity and distance is described as a significant part of in-patient nursing care [53]. This balance allows nurses to act in a professional way [54]. Therefore, they should be able to establish a close relationship with the person and at the same time look at this relationship from a distance [54].

Chronically ill persons are not only in need of functional nursing interventions but also require support for the work of coping and adjusting during the entire course of disease [51]. This poses an additional challenge. Furthermore, it is important that nursing care for chronically ill persons is focused on the entire course of disease and addresses the complexity of a chronic disease [51]. With regard to the situation examined in this study, this requires adjusting care to the progressing course of multiple sclerosis. There is a high need of support for the coping process, particularly after an exacerbation. The exacerbation caused a change from a stable stage to a deteriorating stage of chronic illness [55]. Symptoms were no longer controllable, and the affected person lost physical abilities. It was necessary to adjust activities of daily living to a new situation [55]. As the woman was confronted with progressing physical impairments and therefore became increasingly dependent on nursing support, she experienced a crisis. During this time, the need for support and for adjusting to the new situation was high. However, the woman failed to adapt to this situation and to return to stability [55]. So, she decided to prematurely end her life.

The particular role of chronically ill persons in society can also be challenging for nursing care [51]. Nurses must be aware that chronically ill persons experience an ambivalence between being ill and being healthy. It is necessary to pay increased attention to this ambivalence [51]. Additionally, this ambivalence is associated with the desire for autonomy [51]. Thus, nurses should be able to concentrate not only on patients´ deficits but also on their resources [51]. Schaeffer and Moers (2000) describe the necessity of this rethinking as "accompanying and supporting persons on *their* way to resuming and maintaining wellbeing and an autonomous way of living "(S. 476) [51]. In the situation described in the current study, the affected person's will for self-determination was of central importance. For the nurses, the wish of the affected woman was paramount, and they regarded it as their task to support her on her way, irrespective of their personal attitude. This is in accordance with a study by Mattiasson and Andersson (1994). The authors came to the conclusion that nurses caring for persons with the wish for premature dying respect the patient’s will for autonomy even if this is challenging for them [56]. However, to respect patient autonomy, an accordance of nurses' and patients' aims is not necessary, as Boppert (2002) emphasizes [57].

It is evident that caring for chronically ill persons in general entails many challenges [51]. In the current study, VSED posed an additional challenge since the agents were unfamiliar with this method [58]. According to Knight (1921), uncertainty arises in situations in which behavior cannot be traced back to a person’s own opinion or to scientific information [59]. Related to the given situation, Knight's statement can be confirmed. The behaviour of all agents was characterized by uncertainty since they had neither experience nor expertise concerning VSED.

### Significance of personal attitude

In the professional context, a personal attitude results from a habitus [60]. A person’s habitus represents her or his patterns of perceiving, thinking and acting [60]. All experiences of a person are expressed in the habitus which is imprinted by the position a person holds in society [60]. Bourdieu's description of habitus allows to explain why the opinions of various professions and relatives are different. All agents have various experiences and hold different positions in society. This has probably resulted in developing different personal attitudes towards VSED. The results lay bare that the following aspects are relevant for adopting a personal attitude: one’s own experience, prior knowledge, faith and role as well as the performing person's age, disease and deliberate communication of VSED. A study by Harvath et al. (2004) revealed that nurses mainly adopt an approving attitude towards VSED and are willing to accompany persons during VSED [61]. In the current study, nurses also showed an affirming attitude towards VSED. Therefore, it could be concluded that the nursing role is associated with an affirmative attitude towards VSED.

A person’s attitude towards death is influenced by personal, cultural, philosophical and social belief systems [62, 63]. This is in accordance with the statements of the participating nurses in the current study, expressing that their attitude towards VSED is associated more with culture and faith than with the age and education of a nurse.

### Dealing with concerns and fears in a professional way

All agents expressed that VSED as an unknown challenge induced fears and concerns. In the literature caring for dying persons is described not as a professional but a personal challenge [64]. Nurses must reflect their emotions concerning their own mortality and at the same time they have to take over the care for dying persons in their professional life [64]. In the current study, nurses already were experienced in caring for dying persons. However, VSED was unknown to them and raised fears and concerns. These fears were related to interventions against the sensation of hunger and thirst as well as to the following questions: What would happen if the affected person decided to resume food-intake? Is the dying process in VSED different form the normal dying process? How should nurses communicate VSED towards external persons?

Harvath et al. (2006) reported that several nurses caring for persons with a wish for premature dying felt personally responsible for this wish and tried to dissuade them from the way they had chosen [22]. The present study cannot confirm this result. Participating nurses were able to distance themselves clearly from the woman's wish and assigned the responsibility to her. Additionally, Harvath et al. (2006) mentioned that nurses expressed fears about offending the law by caring for persons with the request for premature dying [22]. In our study, fears concerning the legal situation also arose. Nurses reported being uncertain since the general manager accused them of doing something illegal. Another fear-inducing factor in this context are imaginations of letting somebody die of thirst in an excruciating way [26]. The participating nurses shared this fear.

To cope with situations causing uncertainties, fears and concerns, nurses have to achieve a balance between their personal and professional ethics and patient autonomy [22]. In that respect, the participating nurses expressed the need for expertise and a professional contact person for VSED-related issues. Furthermore, knowledge about the legal situation is also important to reduce fears. Having gained positive experiences with caring for a person with VSED also proved to be helpful for the nurses in order to reduce fears in the future.

### Limitations

For the first time, this study proposed a central model of caring for a person during VSED in a long-term care institution. We derived this model from the case underlying this study. Due to the degree of abstraction, it might be assumed that the theoretical model generated in this study can be transferred to the in-patient setting. However, this should be tested.

### Implications for practice and research

This study reveals the need of professionally embedding VSED into practice. To ensure that VSED can be systematically available as an additional option to prematurely induce death, educative interventions and quality controls are necessary. Since VSED is a complex phenomenon, it is required to involve palliative care into practice development early on and comprehensively. Furthermore, it is necessary to distinguish between accepting VSED and respecting the wish for it. With regard to performing VSED, the study lays bare that consensus, information and moderation are indispensable for the team. To facilitate dealing with VSED in practice, this method and its possible complications require further research. This study offers a conceptual model that should be verified by means of a hypothesis-testing approach. Further research ought to consider different situations for performing theory-generating studies. It is recommended to use lifeworld approaches to investigate experiences of the agents involved. Based on the results, interventions for health care professionals and relatives should be elaborated. Additionally, clinical guidelines ought to be developed to professionalize dealing with VSED in institutions.

## Conclusions

The elaborated model of caring for a person during VSED in a long-term care institution allows health care professionals to reflect VSED in an in-depth and professional way. The results show that the problem in institutions does not consist of caring for a person during VSED. It is rather associated with different attitudes towards VSED probably resulting in conflicts.

The personal attitude towards VSED is influenced by one’s own experiences, previous knowledge, role and faith as well as by the performing personsˊ age, disease and deliberate communication of VSED. If the agents involved are conscious about these influencing factors, they are able to reflect their attitude and to deal with VSED in a professional way. Developing an attitude towards VSED in an institutional and personal way is indispensable.

Thus, it seems to be important that long-term care institutions get acquainted with the option of VSED and explain their position towards it. This comprises obtaining information concerning scientific knowledge and the legal situation. In this way, the institution and every employee can develop a professional attitude towards options of premature dying. If a resident requests VSED, the institution is prepared to meet this wish in a professional way and to offer advice. Relatives may also benefit from a professional attitude towards VSED since they experience a challenging time. They are confronted with the physical decay of their family member and have to cope with fears. Professionally handling VSED paves the way for a dignity-preserving final lifetime for the affected person and the relatives.

## Abbreviations

DIGNITAS and EXIT: Swiss legal assisted suicide organizations
EKSG: Ethics committee of the canton St. Gallen
MAXQDA: Software for analysis qualitative data
VSED: voluntary stopping of eating and drinking
